# Driving forces in the origins of life

**DOI:** 10.1098/rsob.200324

**Published:** 2021-02-03

**Authors:** K. A. Dill, L. Agozzino

**Affiliations:** ^1^ Laufer Center for Physical and Quantitative Biology, Stony Brook University, Stony Brook, NY, USA; ^2^ Department of Chemistry, Stony Brook University, Stony Brook, NY, USA; ^3^ Department Physics and Astronomy, Stony Brook University, Stony Brook, NY, USA

**Keywords:** origin of life, survival of the fittest, autocatalysis, fitness

## Abstract

What were the physico-chemical forces that drove the origins of life? We discuss four major prebiotic ‘discoveries’: persistent sampling of chemical reaction space; sequence-encodable foldable catalysts; assembly of functional pathways; and encapsulation and heritability. We describe how a ‘proteins-first’ world gives plausible mechanisms. We note the importance of hydrophobic and polar compositions of matter in these advances.

## Graphical abstract

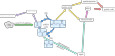

## 1. What forces drove the origins of biology?

How did life begin? What drove the transition, more than 3 billion years ago, from physical chemistry to biology (Pchem_2_Bio)? We seek the origins of biology’s forces of sustainability and persistent innovation. To be clear, this is not the same as seeking mechanisms of self-replication. Here is a metaphor. Consider an imaginary self-replicating mouse trap. This device is outfitted so that it can reach into a bin of metal and wood parts and assemble a copy of itself. But what happens when the bin runs out of parts? Self-replication, by itself, is not a sustaining force. Nor does it explain how it’s self-replication abilities arose from physico-chemical stochastic processes in the first place. Here, we are interested in the causative actions that could have driven physical chemistry (Pchem) to discover biology (Bio), with its unique abilities to propagate in ways that are resourceful, adaptive and persistent.

First, an overview of related research. The origins field has a long history, dating back, at least, to Darwin’s idea in 1871 of a ‘warm little pond’ [1,2] and then of a ‘primordial soup’ [3,4]. Many are studies of prebiotic chemistry, including prominent early ones by Urey [5] and Miller [6] in the early 1950s, and Orgel in 1968, [7], which have sought molecules and conditions that were plausible on the early earth and their possible reactions. Others have focused on what biological precursor molecules might have come from space, for example, in the Murchison and other meteorites [8]. There have been speculations on chicken-and-egg ‘what-came-first’ problems. Metabolism first [9]? Proteins and functionality? Nucleic acids and information? An RNA world first [10,11]? A world of encapsulated replicating RNAs [11]? A lipid world [12]? What interactions might have led to the genetic code [13–15]? For general reviews, see [16–19]. And since there are no definitive experiments yet, much work is speculation using theory and modelling, such as of primitive replication, in Eigen’s quasi-species models [20,21], the GARD model [22] and others [23–25]. The present work is aimed in a different direction: to seek plausible origins of biology’s drive towards persistence and long-term innovation. Here are our starting points.

## 2. Our premises about prebiotic chemistry

— **Life originated on Earth.** We assume that origins happened on Earth. While amino acids and simple organics are found on meteorites from space, we are interested in more life-like complexity, which is unlikely to have come from *panspermia* (i.e. originating in space before coming to Earth [26,27]).
— **Life arose by natural laws,** including chemical transformations of simpler molecules into more complex ones as well as physical processes such as diffusion, binding, catalysis, chemical reactions and changes in molecular concentrations and conformations.
— **Like today, it was far away from equilibrium.** Life is a non-equilibrium (NEQ) state. It requires continual input of energy and matter. Earth’s energy input from the sun is huge [28]. At some point during life’s origin, some chemical reactions became linked with energy to drive them. Chemistry ‘learned’ to harness energy, through gradients of ions or protons, or daily cycles—of light and dark, or heating and drying, or changes in salts, temperature, or redox or pH states, for example.
— **It started with simple chemicals, maybe in a special environment**, like a prebiotic soup, a shared space, maybe ‘Darwin’s warm little pond’ [2] or a hot hydrothermal vent in a sea floor. That medium contained prebiotically plausible simple molecules, such as methane, ammonia, water, some amino acids and nucleic acids, catalysed by surfaces, minerals and metals [3,4].

## 3. Distinguishing between life and non-life

To scrutinize the transition, we first ask what distinguishes living from non-living systems. Living systems metabolize (i.e. take in resources), grow and duplicate. But some nonliving systems also do these things. Candle flames can take in fuel, oxidize it, grow bigger fires and light new fires. Oil droplets can grow and duplicate. Related processes occur in self-replicating computer codes or in human institutions that compete for resources. For our purposes, a living system:

— is ‘wet’ (i.e. made of molecules);
— has units of agency, such as cells;
— metabolizes, taking in matter and energy;
— grows and replicates independently; and
— has lineages and heritable variation.

This definition excludes fires and oil drops (no heritability), viruses (no independent growth) and self-replicating computer codes or human institutions (not wet). It includes plasmodia, which are multi-nucleated but bounded. Others have defined life to include Darwinian selection [29] or computer codes such as Artificial Life [30–32].

### 3.1. The dynamics is different: persistence versus relaxation to equilibria

The biological dynamics we consider is evolutionary change. Both living and non-living matter have dynamical behaviours that entail stochastic searching of degrees of freedom (DOF), sampled by the actions of random forces and driven toward macrostates that can be predicted by a variational principle (the second law of thermodynamics in physical systems; *survival of the fittest* in biology). But the details are very different; see table 1. For one thing, biology’s evolutionary tendencies are not a drive toward equilibrium. For more than 3 billion years, life has been in a stable non-equilibrium. Survival of the fittest (SOF) is a principle of long-term sustained dynamics, not equilibrium. For another thing, different dynamical processes dominate biological evolution versus chemistry. In Pchem, atoms and molecules search positions, velocities and conformations, sampled by random thermal forces. In biology, cells search different growth rates, sampled by random changes in monomer sequences in proteins and nucleic acids. And, the nature of disorder is different; their corresponding entropies do not even have the same units. How did Pchem come upon, and enable, biology’s processes and forces?

**Table 1.** Dynamical processes in biological evolution are different than in physical chemistry.

	thermal physics	biology
actors	atoms, molecules	biochemical reactions
degrees of freedom	coordinates and densities	chain sequences of monomers
search space	molecule physical states	chemical types and reactions
driving principle	2nd law	survival of the fittest
tends toward	equilibrium	self-sustaining

## 4. Survival of the fittest is a persistence principle

### 4.1. Evolution is sustained by positive feedback

What is the nature of SOF as a dynamical variational principle? Much of textbook physical chemistry describes systems subject to negative feedback: they are stable, subject to restoring forces, having states of equilibria to which they return after perturbation. By contrast, the centerpiece of biology’s evolutionary dynamics—SOF—is a principle of *persistence*, i.e. a sustained capacity for a particular type of positive feedback^1^ or what, in simpler chemical systems, would be called *autocatalysis* [33,34]. One example of autocatalysis is *A* + *B* → 2*B*. Another is a forest fire, where burning is cooperative among fuel elements that are at high density. Here, we refer to this positive feedback as *bootstrapping*, taken from the expression: *lifting yourself up by your bootstraps*.^2^ Biological evolution is sustained by SOF. What physical chemistry begat that principle?

### 4.2. The SOF principle, described in general terms

Suppose you have some operational device that has persistent input and output; for example, a cell, a machine or a company. You can tweak the inner workings of the device to alter its productivity. *Fitness* is a measure of how effectively (by some metric) the input resources are converted to output. A company can tweak its process to make more product from less resource. In this context, *survival* measures the amount of input resource the device takes in. If a company makes product more efficiently, then the company gains a bigger market. This gives it access to even more resources, allowing it to outcompete other such companies for resources. In SOF, there is a feedback loop: *advantageous actions* are rewarded by new capacity to take *more actions*. The better the performance, the greater the access to even more resources, creating a virtuous cycle of improvement and dominance over the resource pool.

### 4.3. Biology implements SOF in a specific, clever and convoluted way

The pawn that the hand of evolution moves is not the cell, but cell lineages. The metric of survival is the population of a cell lineage relative to others. The ‘knob’ that evolution turns to change that population is the growth rates of cells. Evolution ‘turns that knob’ by random mutations of proteins (and also recombination, lateral gene transfer, plasmids and gene duplication today). A cell’s growth rate is largely determined by its rate of protein production. Hence, here is how the SOF positive feedback loop is implemented in biology: a change such as a mutation increases a cell’s growth rate, causing the cell to duplicate faster, increasing the population of that cell’s lineage of ancestors relative to other lineages. This gives that cell’s lineage greater access to resources in the next generation. This positive feedback principle leads to some of biology’s most marvellous features, described below.

### 4.4. SOF acts by *advantages,* not by *averages*

Positive feedback processes can be controlled by small fluctuations. Compare to a river. A river’s flow properties are dominated by the largest and deepest channels, not the small tributaries, because the typical observables are averages, which are dominated by the biggest flows. By contrast, a key feature of positive feedback is that it can become dominated by the very smallest metaphorical tributaries, provided that those flows are somehow advantageous to the process [35,36]. It allows for ratcheting of advantage. It raises up winners: the few and the good can bootstrap up to dominate over the many and the average. If a single individual cell happens to be well fit for its environment, it grows rapidly. Its lineage can come to dominate the population. This positive feedback manifests as adaptability, innovation, improved match for environments and apparent goal directedness. We note that once an improvability process such as SOF is discovered, there are no limits to the marvellous intricacies it can lead to.^3^

In the Pchem_2_Bio transition, how did stochastic physical dynamics ‘discover’ stochastic biological dynamics? How did polymer chain sequences emerge as the searchable degrees of freedom? What random processes searched and sampled them? And what autocatalytic chemical or physical process could have bootstrapped its way to becoming cellular SOF? Below are four important ‘discoveries’ that Pchem made to reach biology, three of which are positive-feedback bootstrap processes.

### 4.5. Pchem_2_Bio in steps

Consider Pchem_2_Bio as a kinetic process. We are free to divide the average pathway into two sequential steps, real or conceptual, since we can arbitrarily choose the barrier heights, one of which could be zero. The point of division into two steps is to help elucidate the mechanism. The second kinetic barrier, the final step to biology, as defined above, must have had all ingredients present: proteins for function, RNA or DNA for information, and encapsulation and metabolites. But two-state kinetics gives no mechanistic insight; it happens as a single event. Keeping in mind the primacy of understanding driving forces, we postulate below a prior step: proteins develop primitive functions before RNA and proteins together create a genetic code. We argue that protein folding offers a driving principle.

In this view, the first step is amino acids becoming linked into short random peptides by Pchem processes, catalysed by surfaces or metals, for example. Proteins grew longer and catalytic through an autocatalytic foldamer-catalysis process (*the foldcat bootstrap*), generating a diversity of actions. Proteins and metabolites assembled into primitive biochemical pathways, through *the catpath bootstrap*. This results in a stable community of molecules, a *nearbiotic soup*. This soup, however, does not satisfy our definition of a system that is live. Rather, this is just a non-equilibrium chemical intermediate state along the way.

In the second step, the nearbiotic soup could then divide into compartmentalized units of individuals (i.e. proto-cells) that could compete for resources. Those units have heritability, encoded in informational memory molecules, defining lineages on which SOF can act.

## 5. Major discoveries in Pchem_2_Bio

Below we list key discoveries made by physico-chemical processes on the road to biology. (1) *Coupling drivers to chemistry.* Non-equilibria (NEQ) sampled and drove chemical reactions and molecular processes. (2) *Proteins as mobile programmable catalysts.* Monomer sequences in proteins became searchable degrees of freedom, giving programming catalysts and molecular machines. (3) *Assembling biochemical pathways.* Functionally similar reactions associated into spatially localized pathways. (4) *Creating individuals and lineages.* Encapsulation into cells allowed for a distinction of SELF and competition. A genetic code, memory and heritability allowed for survival of the fittest. Our proposition here is that they needn’t have happened all at once. A first step of (1)–(3) would require only proteins. Even today, the existence of horizontal gene transfer implies that linear heritability is not an obligatory early step.

### 5.1. Dynamical processes can sample and drive molecular processes

Was there some special aspect of dynamics in general that created or enabled life [38]? We consider two roles of dynamics, *per se*, in origins: (i) as a mixer and random driver of chemical reactions, and (ii) through *specific mechanisms* that can drive particular relevant innovations.

#### 5.1.1. Forces of disorder can explore chemical reaction space

In general, NEQ *per se*, is not a driver towards order. The sun, winds, waves and volcanoes drive randomness, mixing and disorder. Even so, disordering can give predictable outcomes. For example, thermal forces that randomize the velocities of gas atoms lead to the ideal gas law, a precise relationship. But the randomization that matters on the road to biology is over a very different space than that of gas velocities; it is over the space of chemical reactions. Early earth dynamics could drive different molecules together randomly, sometimes reacting with each other, sometimes catalysed by surfaces, and continually producing product wherever there are continual inputs of appropriate energy and matter [39].

**Figure 1.**
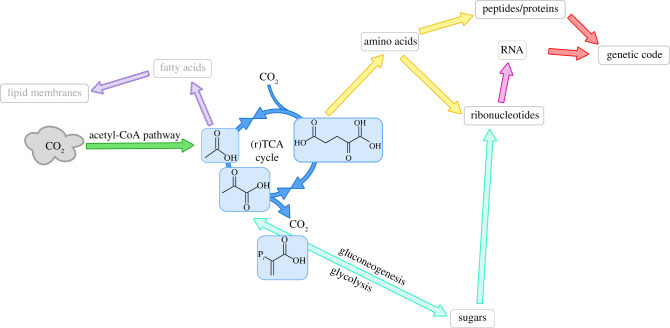
Overview of the biochemistry of living systems. These processes at the core of life are relatively simple, few and coupled. Figure adapted with permission from [41].

And although organic-molecule reaction space is very large [40], the space of today’s biochemical reactions is relatively small and simple [41] (figure 1), hence ancestral versions of them must have been similar [42,43]. There is no reason to believe there was a specific goal-driven force to select out those reactions that would become biochemistry. But geophysical mixing dynamics could at least have searched and sampled some simple reactions, which, through particular dynamical mechanisms described below, could have led to biology.

#### 5.1.2. Far from equilibrium drivers toward persistence and innovation; not just restoring forces

Prigogine and colleagues popularized the view that biology-like spatio-temporal patterning—in chemical oscillators like the Belousov–Zhabotinsky reaction, for example—can arise from NEQ processes [44,45]. Non-equilibrium forces are special; they differ in at least two ways from equilibrium forces.

First, non-equilibrium forces are zero at equilibrium. For example, while bar magnets have a static pull, electromagnets have no pull when the electric field is turned off. In Fick’s law, particles stop flowing when there is no concentration gradient. Also, hurricanes operate only when the underlying thermal conditions drive them. Non-equilibrium structures and organization are sustained by non-equilibrium inputs of matter and energy. Second, NEQ differs by push versus pull, i.e. by supply versus demand. Near-equilibrium processes are *pulled* toward equilibria, a tendency *towards* a state of minimum free energy. They are governed by the second law of thermodynamics. By contrast, FFE is *pushed* by input energy and matter that are out of equilibrium. Imagine a flood that carves a new river bed; it does not aim to go any particular place, it just pushes water, which flows through a path of least resistance. Evolution does not steadily march towards predetermined goals [46], like second law equilibrium restoring processes do.^4^ The NEQ realm is broad and innovative, through particular mechanisms, many of which are not yet fully understood, and two of which are described below.

### 5.2. The foldcat bootstrap: protein foldamers as *programmable* catalysts

#### 5.2.1. The importance of proteins as programmable catalysts

Biology would be impossible without its machines and catalysts, protein enzymes. On the one hand, Orgel and others argued that there is severe difficulty in achieving biochemistry-like reactions with only prebiotically available catalysts [18,47]. On the other hand, important recent experiments have achieved significant reactions using prebiotically available catalysts [48–52]. Even so, chemistry in the prebiotic era was hostile to chemical innovation. The catalysts for those reactions were mineral surfaces or metal ions, many of which were spatially immobile (not accessible to substrates), capable only of catalysing limited reactions, each only under limited and different conditions, and only where substrates were sufficiently concentrated.

Biology is more innovative than prebiotic chemistry. Biology’s catalysts—mostly proteins—are mobile and can go where the substrates are; can be altered to work in different environments, including just in water, or in membranes; can operate at whatever ambient temperature is needed for the organism; and are readily tunable to any degree that is needed to fit within whole reaction pathways and cycles. Protein catalysts could be called *programmable*, in the sense that their extraordinarily wide range of capabilities can be controlled by just a simple single kind of process, namely mutating amino acid sequences.

This importance of this breakthrough—of discovering programmable catalysts—can be illuminated with a metaphor. Compare a fictitious prebiotic organic chemist ‘demon’ (i.e. working with random processes) to a corresponding biology demon. The Ochem demon cannot create a complex multi-step process without many different specific catalysts, each chosen for different conditions, some with intermediate products produced in particular ways. This is sufficiently challenging that academic organic chemists can publish research papers about them! By contrast, the Bio demon just spins some dials on a big dashboard, picking a reaction type, picking the solvent and temperature conditions, picking the desired acceleration and linking multiple reactions together by stringing together pathways of multiple enzymes. Of course, much trial and error is needed for both demons. The early discovery, by physical chemical processes, of catalysts that are explorable and optimizable through random changes of sequences of monomers in a polymer chain is arguably one of the most important steps made during the origins of life because of its capacity for rapid trial-and-error invention of complex chemical processes and diverse functionalities, all brought together under single conditions. Our term ‘programmability’ here does not refer to heritability or a genetic encoding; rather, it is simply intended to express that changing an amino acid sequence can change a molecule’s functional capability.

Here, we describe a mechanism for the origins of proteins as programmable catalysts, controllable through their amino acid sequences. We call it the *foldcat bootstrap mechanism*. It is an autocatalytic process by which short peptides become elongated, sequence selective and develop primitive versions of the today’s protein enzymes and machines. It addresses the following question: what physical process might drive particular subpopulations of chain sequences to self-amplify at the expense of other subpopulations? In this mechanism, random peptides fold and help catalyse the elongation of others in a primitive ribosome-like way. In this way, short-chain peptides grow longer and more plentiful, growing protein mass.

**Figure 2.**
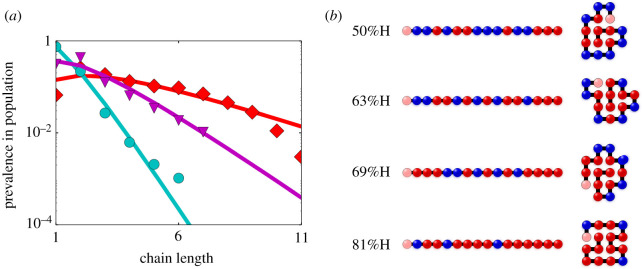
(*a*) The Flory problem. Typical polymerizations create mostly short chains. Longer chains are exponentially less populated. (*b*) The HP model of foldamers. H(ydrophobic) are red, P(olar) are blue. Different HP sequences fold to different native structures. HP sequences fold in ways that lead to maximal burial of the H monomers into a core, minimizing contact with water. Figures reproduced from [53].

There are many plausible prebiotic processes that can polymerize individual amino acids into peptides, or nucleic acids into short DNA or RNA molecules. But these polymerizations all suffer from the so-called *Flory problem*, namely that the resultant chains are mostly very short (≈2–8-mers); longer chains are exponentially less probable (figure 2*a*).

Known prebiotic polymerizations also do not address (i) how the randomness in polymerized sequences leads to ordered and informational sequences, and (ii) how such processes became *autocatalytic*, leading to stable steady states of production of long-chain informational-sequence polymers.

The *foldamer catalyst hypothesis* [53] offers an explanation. In this hypothesis, chains are polymerized using two types of monomers: hydrophobic (H) and polar (P), as modern-day proteins are.^5^ When H and P monomers are linked into long chains, like today’s proteins, different HP sequences spontaneously fold in water to different ‘native’ structures [55] (figure 2*b*). The structures are driven by the oil–water principle that hydrophobic monomers seek to minimize contact with water.

**Figure 3.**
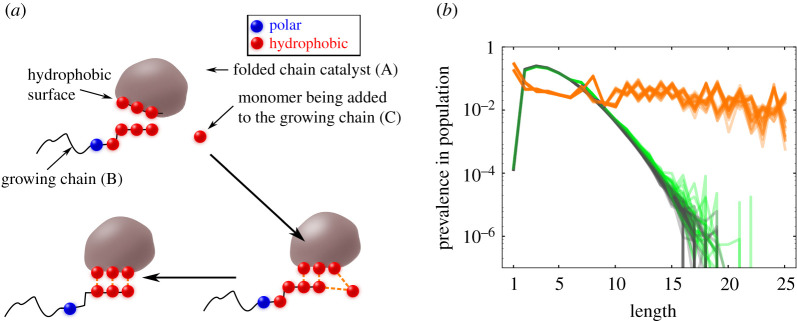
(*a*) The blob chain elongates the string chain. It folds, and has a landing pad, putting the string chain next to new monomers, thus elongating the second chain. (*b*) This foldcat mechanism (orange) bootstraps to longer-chain populations, overcoming the Flory length problem (green). Figures reproduced from [53].

According to this hypothesis, some short-chain HP sequences will compactify in aqueous solutions into structures that have some exposure of their hydrophobic residues on their surface. Call those hydrophobic surfaces *landing pads*, and those chains *catalysts*. If a second short peptide chain lands its own H monomers on the sticky hydrophobic surface of the first one, a catalyst, then the second chain will undergo an enhanced rate of covalent elongation because of the sticky localization of the chain and an H monomer to be added (figure 3*a*).

**Figure 4.**
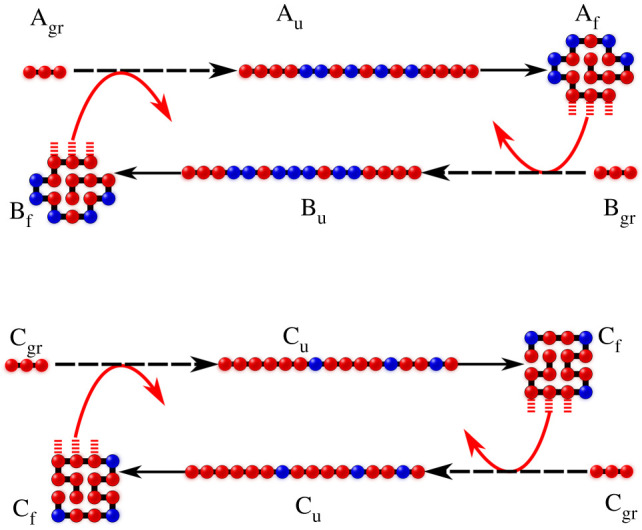
Only a small subset of all HP sequences form an autocatalytic set, propagating themselves, at the expense of other sequences. In this way, random short chains become informational longer chains that populate their volume in steady state. Figures reproduced from [53].

The HP foldcat mechanism gives the three properties sought above. First, exact enumeration in the HP lattice model shows that this mechanism leads to amplified populations of longer chains (figure 3*b*). It also leads to reduced subspace of HP sequences, initiating a process of converting random sequences to informational polymers. And, it generates an autocatalytic set that continues propagating other sequences in that set; see figure 4. The following paragraphs give arguments for the plausibility of this mechanism.

#### 5.2.2. Evidence for folding in HP polymers

Today’s protein folding code is dominated by the binary HP patterning in the sequence [53,55]. This is proven in experiments where proteins that have been massively mutated, in ways that preserve only a given HP pattern, still fold to their appropriate native structures [56–59].^6^ Moreover, HP foldability does not even require that the polymer backbone be a peptide. *Peptoid* chains (polymers of N-substituted glycines) can also fold into HP-sequence-dominated structures [60]. Further evidence for the early role of hydrophobicity is that ancestral proteomes are more hydrophobic [61,62].

#### 5.2.3. Catalysis and binding are ubiquitous in peptides and proteins

Functional peptides are ubiquitous in today’s biology (the *Handbook of Biologically Active Peptides* [63] is more than 2000 pages long!). Short proteins function as hormones, signalling molecules, growth factors, venoms, antibiotics and more Enzymatic activities are known in chains even as short as dipeptides [64–66], and including ATP binding activity [67]. 7-mer amyloid peptides can catalyse reactions and auto-catalyse their own formation [68,69]. So, amyloid structures might have been prebiotic catalysts [70]. Moreover, proteins are highly promiscuous binders. For example, half the yeast proteome has protein–protein binding affinities stronger than 1 kcal mol^−1^ [71]. And regarding whether simple peptides could help elongate others, we note that non-ribosomal peptide extension and chemical modification is done on peptide scaffolds [72]. Furthermore, once a protein has a binding site, that site often readily mutates to become an active site [73].

#### 5.2.4. Perspectives on the foldcat mechanism

Here, we note some caveats and suggest some experimental tests. First, we are not aware of any evidence yet for simple peptides folding and catalysing chain elongation in other peptides. But we are also not aware of any tests of it. The value, as we see it, in the present theoretical speculation, is in giving a mechanism that is sufficiently detailed that it can be tested through experiments.

Second, what are the limitations of the model? While figures 2–4 illustrate the foldcat mechanism with graphic simplifications—to two dimensions, to a code that is only binary (H and P), and to conformations that are confined to a lattice—extensive studies with larger code alphabets and in 3D [56,57] have shown that this simple model recapitulates important behaviours of real proteins. The 2D HP model has its equivalent of secondary and tertiary structures; the thermodynamic behaviours of short chains in 2D resembles longer chains in 3D because of the dominance of surface-to-volume ratios and hydrophobic interactions; and as noted above, the sequence-to-structure code degeneracy in real proteins is known from experiments to be close to binary [55]. For understanding the nature of both conformational and sequence spaces, microscopic atomic details often matter much less than an ability to do coarse-grained enumerations, which is readily done in simple models. At present, it is not possible to draw unbiased inferences about the nature of sequence space with more atomistically detailed models than HP lattices. And, while the mechanism illustrated here adds only H monomers, driven only by hydrophobicity, this is just an illustration because any broader distribution of amino acids that would have been used in primitive proteins would have likely harnessed additional interactions as well.

Third, while the example above of the Foldcat mechanism illustrates ‘inventing’ primitive ribosomes, it also follows that there would be broad random coverage of sequence-structure space, so other (weak) protein machines would be generated too. We infer that proteins and functional diversity could have been a first step in Pchem_2_Bio, followed by encapsulation, heritability and memory.

### 5.3. The *catpath mechanism* assembles functional pathways

Imagine the prebiotic stew above, of small molecules and catalysts. How could that stew have been divided up and encapsulated into individual cells? Physico-chemical actions would only aggregate them together randomly into vesicles or droplets. That would not lead to biology. Each cell needs assemblies of reactions that form functional pathways, cycles and hypercycles (i.e. interlinked cycles)? What would cause different enzymes with related functions to come together in space, like bucket brigades, in which the output of one reaction is close enough to become the input of another reaction? Here, we describe such a process.

The *catpath mechanism* is a non-equilibrium reaction-diffusion mechanism that brings reactions together in space based on their related functionalities [74]. In this process, a catalyst *A*, fixed at a given location, draws a catalyst *B* in its spatial neighbourhood; the effective attraction between the catalysts (*cats*) is mediated by a common substrate or product, on which they both act.

**Figure 5.**
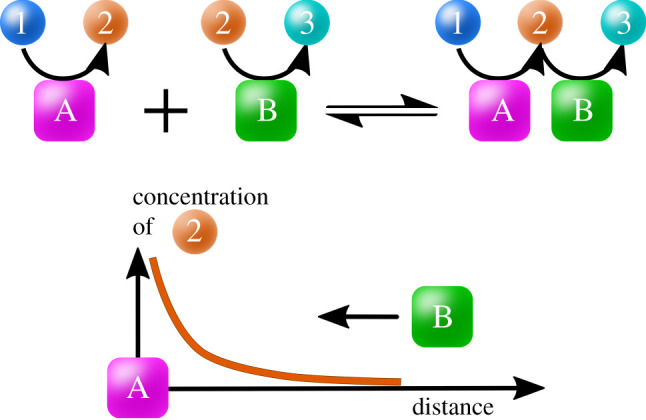
The catpath attraction: one catalyst, A, attracts another, B, mediated by a common substrate. A converts 1s to 2s. B converts 2s to 3s. In steady-state, S produces concentrated 2s, which bind to B, attracting B to A.

Figure 5 (top) shows the catpath mechanism. The square-box objects in the figure are catalysts, such as enzymes. The letter inside each catalyst box is an identifier of the reaction it catalyses. The catalysts are mobile and free to diffuse, towards or away from other such catalysts. The circular objects are the substrates and products, typically small molecules. Inside the circles are numbers that identify or label them. The arrow in each icon shows the direction of catalysis, from substrate to product.

In the catpath mechanism, a mobile catalyst molecule B, which converts 2s to 3s, diffuses toward the position of a catalyst molecule A, which converts 1s to 2s; see figure 5 (bottom). This attraction is a *reaction–diffusion* process [75]. Because the A cats are continuously supplied with 1s, so they continuously produce 2s. These product 2s will diffuse away from the parent A at some rate, but will concentrate around A for certain relative speeds. The B cats have a binding affinity for their substrates, 2s in this case. So Bs will diffuse toward the 2s, thus toward the A cats. In this way, A and B cats are attracted to each other, mediated by a small molecule substrate/product in common.

#### 5.3.1. In the catpath mechanism, *function dictates structure*

**Figure 6.**
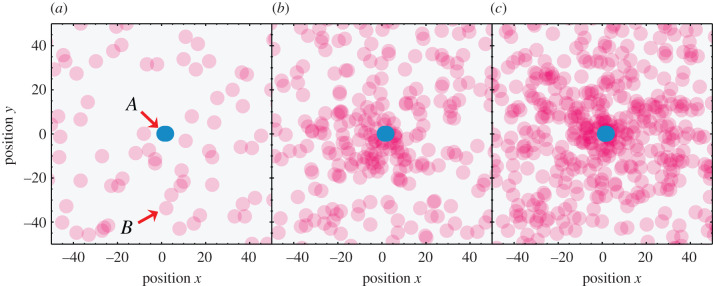
Simulations of the catpath mechanism shows that enzymes A and B can attract each other if they have a substrate or product in common (from C. Kocher, L. Agozzino & K.A. Dill 2021, unpublished data; and [74]). This non-equilibrium force can drive assembly of functional pathways.

The catpath process contrasts with two standard situations: (1) two independent particles will simply diffuse away from each other, or (2) two particles with mutual affinity will come together and bind each other. The catpath attraction is not based on a binding affinity, A–B; rather, it is an example of *function driving structure*^7^: processes that have a common mediator come together. Unlike simple A–B binding affinity, catpath is a non-equilibrium force; there is no attraction unless 1s are continuously supplied. It is driven only by the commonality of the small-molecule agent that is the product of one cat and the substrate of the other. We note two additional points. First, the catpath mechanism is not unique to protein catalysts, and would also apply, for example, to RNA catalysts. Second, the catpath mechanism bears some resemblance to, and might have been a molecular precursor to, chemotaxis in bacteria [76] (see figure 6), when the due distinctions are taken into account [77,78].

#### 5.3.2. The catpath mechanism could assemble transducers and machines

**Figure 7.**
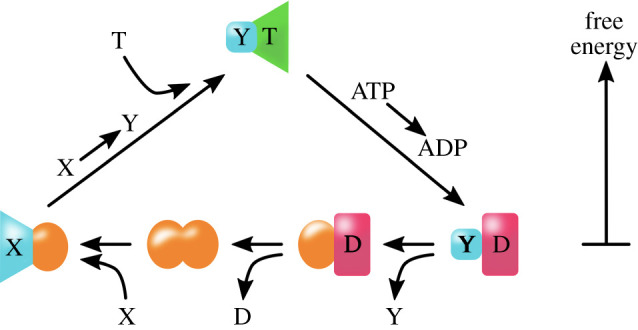
An essential process in origins of biology: couplings that drive uphill chemistry by downhill energy changes. (*a*) Downhill conversion from ATP to ADP can drive uphill processes like moving molecules uphill against their concentration gradients.

Critically important in biology is energy transduction coupled to chemical reactions. Often one domain of a protein performs an energetically uphill reaction, driven by an energetically downhill reaction in another domain, typically by converting ATP to ADP or by flows of protons or ions down their concentration gradients. Without such coupling, it would be impossible to metabolize food to synthesize biomolecules, to run molecular motors, chaperones, ribosomes or other machines, to perform signalling, or to synthesize biomolecules such as proteins and nucleic acids. Today’s processes are well understood through the physical chemistry of binding events coupled to conformational changes in proteins; see figure 7. These processes, such as in ATPases and GTPases, entail multiple protein domains that are bound together into a complex: one domain performs the uphill action and the other domain converts to ATP to ADP to ‘pay the energetic price’ for the uphill step. A crucial ‘discovery’ during origins of life must have been the combining of two protein domains in such transduction processes [79]. The innovation this allowed, on the road from chemistry to biology, was the ability to power energy cycles and biochemical circuits. Protein domains may have been driven to assemble by the catpath mechanism, but there are no studies yet as far as we know.

#### 5.3.3. The catpath mechanism can drive SOF-like bootstrapping

**Figure 8.**
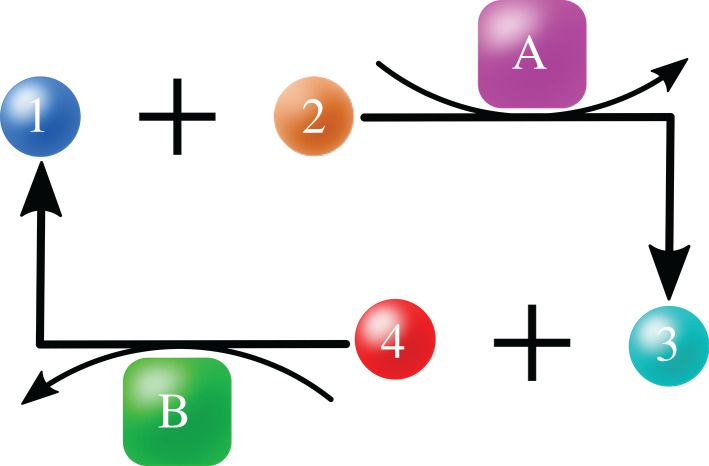
Simple chemical autocatalysis, i.e. positive feedback, based on 2 reactions: 1 + 2 → 3 and 3 + 4 → 1. Swapping in a better A (call it A’) speeds up the cycle, making 3s even faster, further accelerating the cycle, etc.

Where does the SOF principle come from? Might its prebiotic precursor have been some simple autocatalytic chemical cycle, such as shown in figure 8? Here is what we are seeking to explain. If a chemical process is changed in a way that causes it to run faster (in biological language, a mutation increases the fitness), how does that lead the process to recruit more resources for itself (more survival)? For the autocatalytic cycle in figure 8, the catpath Mechanism can link survival to fitness. Catalyst A converts substrate 1s and substrate 2s to product 3s. Catalyst B converts substrate 3s and substrate 4s to product 1s. The two catalysts are linked as a cycle: the head of each reaction is the foot of the other. The substrates and products, 1s and 3s, are common to the two reactions. Mutating catalyst A to a better one, A′ increases the cycle speed. Because of the catpath force, the greater cycle speed drives greater attraction to B of A′ relative to A. The machine A′B is more stable and persistent than the machine AB, hence is the more reliable consumer of new resources.

### 5.4. The heritability bootstrap: replicating the ‘self’

Achieving SOF requires informational linkage between how fast a cell replicates, on the one hand, and the size of the population of its lineage, on the other hand. This requires, first, that living systems come in discrete units, i.e. individual agents such as a cell (call it ‘the self’). This compartmentalization is enforced by lipid bilayers and related boundaries. The cell must contain information about how it achieves its growth speed. And it also requires a mechanism for transmitting information down generations, from parents to daughters. Below, we just make brief points about the physical chemistry of encapsulation and heritability.

#### 5.4.1. Encapsulation distinguishes individuals and lineages, enabling competition

In the origins of life, compartmentalization could have arisen from oil droplets or vesicles in a lipid world [12,80,81]. They readily grow and divide. Droplets or vesicles or containers can grow in proportion to the amount of material inside them, providing the first step in a growth-based SOF mechanism. Natural surface-to-volume forces will cause such compartments to split into two when they get big enough, giving a physico-chemical basis for the divide and replicate aspects of SOF. The interiors of such primitive cells would be concentrated proteins, as in today’s cells. Their growth could come from the foldcat mechanism, for example. It would be interesting to see more detailed modelling.

#### 5.4.2. Genomes implement memory for precise heritability

**Figure 9.**
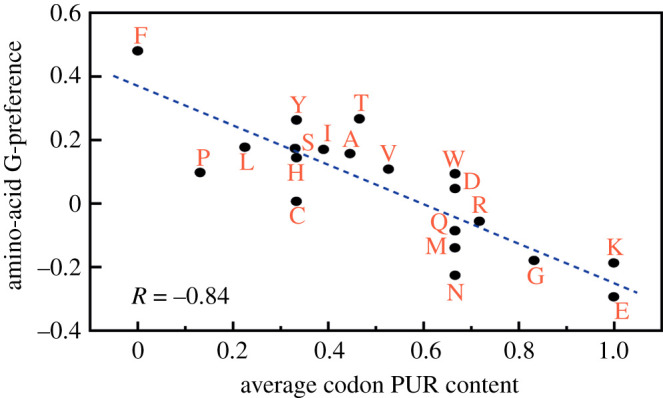
Support for the stereochemical hypothesis. From known structures of protein–RNA complexes, the purine content of the RNA codons (*x*-axis) correlates with the base binding preferences of the amino acid sequences they code for. Shown here is the guanine preference (reproduced from [89]).

SOF requires accurate information transmission: of cell growth rates to lineage populations. This is achieved today by covalent memory in RNA and DNA genomes. A plausible explanation for the physico-chemical origin of the genetic code is the *stereochemical hypothesis* [13,82–86]. In this view, the genetic code arose from weak stereochemical binding affinities between nucleic acids and peptides, ultimately leading to codons and anticodons in today’s more complex machinery. Here are the lines of evidence supporting that mechanism. mRNA coding sequences undergo co-aligned binding to protein sequences [87]. In pyrimidine solvents, amino acids bind to pyrimidine and purine bases in proportion to their hydrophobicities [88]; see figure 9. Nucleic acid base-stacking is driven by hydrophobic interactions and hydrogen bonding [90]; nucleic acids at high concentrations assemble into non-covalent base stacks even without a backbone [91]; free histidine binds an RNA aptamer when selected for affinity [92] and adenine binds to peptide backbones [93]. Evidence of physical affinities also appears in the identity recognition elements by which AA-tRNA synthetases recognize cognate tRNAs [94].

## 6. First a soup of protein machines, then encapsulation and lineages

We have postulated two stages in Pchem_2_Bio: forming a nearbiotic soup requiring only peptide foldamers and metabolites, followed by cellular encapsulation and informational molecules. Here, we give additional context.

### 6.1. Unlike an RNA world, a ‘proteins-first’ world has a plausible sustainability mechanism

The final step to biology, whatever it may have been, would have required all ingredients: proteins, informers, metabolites and encapsulation. But dividing Pchem_2_Bio into two steps, either real or conceptual, allows us to model possible mechanisms in more granular detail. The principal argument here for proteins first is simply that we can identify a possible mechanism. Here, we have argued for the importance and primacy of establishing the driving forces. By contrast, if instead, nucleic acids as a vehicle for memory and information, were to have come first, what would have driven it? We know of no principle or force that would have caused it to happen. Moreover, if memory were first, what machinery would construct it? It is not clear how or why it would construct itself in a self-sustaining way.

### 6.2. Why not an RNA world first?

The idea that origins could have started with RNA came after the discovery by Cech [95] and Altman [96] of ribozymes, namely that RNA can catalyse reactions, making RNA a type of molecule that bridges the folding and function world with the information/genes world [10,97–99]. The RNA-first view has driven many important experiments in prebiotic and nanotech research [100–103].

But the RNA-first idea has some notable difficulties [104–106]. First, RNA just names a type of molecule, and not a driving principle that would sustain it. RNA is useless without a copying machine. Second, proteins are better catalysts [17]. Even where a protein and an RNA molecule can catalyse the same reaction, such as an RNase, which breaks down RNA molecules, the protein version is 100 000-fold better than the hammerhead ribozyme [17]. And RNA-based catalysts are limited, mostly phosphoryl transferases, such as RNA polymerases, ligases and RNA nucleases. The catalytic power of proteins, with 20 amino acids of very different chemical moieties, is much broader than of RNA molecules, with only the four bases, with recognition driven largely by hydrogen bonding.

Third, the most common reaction products from many prebiotic syntheses of small molecules are amino acids, possibly because with only around 15 atoms each, they are easier to synthesize than nucleic acid bases, having around 35 atoms each. And, the yields of the different amino acids in those experiments resembles the compositions in today’s proteins [21]. Fourth, Carter & Wills show that aa-tRNA synthetases came before ribozymes, not the other way around [107]. Fifth, and more importantly, the implication of the Guseva mechanism [53] is that the *foldability of polymer chains* is the crucial ingredient that enables the autocatalytic explosion of functionality in Pchem_2_Bio. Foldability is mainly a property of proteins, not RNA molecules.

### 6.3. Proteins are better for function; DNA is better for information

There is a plausible explanation for biology’s current division of labour in which proteins are functional and DNA is informational. For functionality, you need sequence-structure relations: changing the sequence, changes the structure, changes the function. The physics that enables this is folding. Proteins fold better—and for essentially all sequences—than RNA does. For information, and for memory-like actions, you specifically want the opposite. You want a type of molecule that can store all information the same, with no preferences, with the absolute minimum possible sequence structure relationships. DNA is an almost perfect informational molecule: it is very stiff, has no fold and its double-strandedness protects either strand from binding to external agents (apart of course, from transcription and such.)

### 6.4. A full story of Pchem_2_Bio would entail informers and proteins emerging together

After a nearbiotic soup, the emergence of a genetic code requires both proteins and informational molecules to develop together [108]. Here is evidence for their concurrent development. For one thing, nucleic acids and amino acids can both arise in common from the same prebiotic processes [52]. For another thing, RNA and peptides have binding complementarity, like hands in gloves [86]. So, if a peptide-first world already drives preferences for some peptides over others, it’s easy to imagine them coupling with companion informational molecules. Interestingly, frameshifting at the mRNA/DNA level leads to protein sequences with largely unchanged hydrophobicity profiles [109], indicating how even coarse-grained hydrophobic composition alone, in the absence of specific sequences, could have carried information. In addition, Carter has shown that ‘urzymes’, which are shrunken cores of amino-acid-tRNA synthetase (aaRS) proteins, and which may have been evolutionary precursors, are unstructured small proteins having hydrophobic cores that can work with low-fidelity peptides [110,111]. Carter & Wills have argued that aminoacylated-tRNA molecules must have evolved in parallel with the proteins that they are responsible for helping to make, not preceding them [107].

### 6.5. First, a single happy pond; later, bickering individual lineages

Modelling has suggested that the origins of life started from a single *community in a cauldron*, something like a localized pond, before becoming individual competing cellular lineages, perhaps through an autocatalytic phase-transition-like event [112]. Community in a cauldron as a first step has the advantage that it can be communally supportive since there are no predators yet. The pond doesn’t need to compete, just to survive. Crick speculated [13] that the community-first mechanism explains today’s single genetic code, i.e. that ‘all life evolved from a single organism (more strictly, from a single closely interbreeding population)’. Although there are now counter-examples and non-universality in codes, for example in mitochondria and some nuclear genomes, the differences are small [113].

## 7. Conclusion

By what stochastic physical chemistry did dead matter ‘invent’ live matter? We cannot look to equilibrium principles because life has remained far from equilibrium (FFE) for 3 billion years. Unlike equilibria, which are pulled by goal-like end states, FFE dynamics are driven by the pushing flows of available matter and energy. Fitness is a tendency towards matching to environments, a driver for effective utilization of resources.

What mechanisms might have led to the autocatalysis and SOF? We describe three bootstraps. In the foldcat bootstrap, proteins became controllable catalysts, programmable through their sequences. In the catpath bootstrap, different enzymes come together in space to form pathways. In the encapsulation/heritability bootstrap, biochemistry becomes encapsulated and compartmentalized into cells, and outfitted with genetic memory to link past to future. Proteins and biochemistry, through the first two bootstraps, could have been stably self-sustaining, prior to encapsulation and heritability. Of course, this is presently just a speculation. But, there is no evident alternative mechanism by which nucleic acids could achieve persistent sustainability prior to proteins. A thread through these mechanisms is the antipathy between hydrophobic and polar interactions, in protein chains, in folding, in encapsulation, and in protein-nucleic acid interactions.

## Endnotes

1 That is, a tendency for the leveraging of advantage. We use the term broadly, allowing for genetic drift, where the advantage can be small or in the form of an increased genetic diversity.

2 For our purposes, ‘autocatalysis’ is too specific to chemistry and ‘survival’ is too specific to biological populations.

3 This contrasts with the notion of *irreducible complexity* [37], that living systems are so complex they could not have arisen by random physical processes. Our point here is that an improvability process, even one that operates by trial and error, need not be limited in the intricacies it can achieve.

4 Here is a metaphor from industrial innovation, another process of stochastic discovery: the 60-year trajectory that led to the iPhone in 2007. It started with computers and solid-state electronics in the 1950s. But the drive in the 1950s was not the pull-driven goal to create an iPhone. Rather, the drive was push-driven by commercial opportunities of the day for computers and integrated circuits. The iPhone was just a late-stage consequence.

5 H and P correspond roughly to what Carter calls class I and class II, buried and surface residues [54].

6 Of course, modern proteins use all 20 types of amino acids for both folding and function. This model just indicates that an early code could have been crude, simple and weak.

7 It is the converse of the well-known principle in chemistry and biology that *structure determines function*. Here, function, in proteins, refers to an enzyme’s chemical action, and structure refers to the spatial localization of functional activities.

## Data accessibility

This article has no additional data.
